# Outcomes of Autologous stem cell transplantation in patients with primary refractory Diffuse Large B-cell lymphoma who demonstrate chemosensitivity to salvage chemotherapy

**DOI:** 10.46989/001c.115919

**Published:** 2024-04-10

**Authors:** M Shahzad Rauf, Irfan Maghfoor, Muhammad Aseafan, Khadijah Al Shankati, Ali M. Alhanash, Faateh Sohail, Tusneem A. M. Elhassan, Saad Akhtar

**Affiliations:** 1 Medical Oncology King Faisal Specialist Hospital & Research Centre; 2 College of Medicine, Al Faisal University

**Keywords:** diffuse large B-cell lymphoma outcomes, autologous stem cell transplantation, salvage chemotherapy, Middle East

## Abstract

Rituximab with anthracycline-based combination frontline chemoimmunotherapy can cure 50–60% of patients with diffuse large B-cell lymphoma (DLBCL). However, studies on the outcomes of patients with DLBCL who experience partial response (PR), stable or progressive disease in response to frontline rituximab, cyclophosphamide, doxorubicin, vincristine, and prednisone (RCHOP) therapy are limited, as are data on the outcomes of high-dose chemotherapy (HDC) and autologous stem cell transplantation (ASCT) in patients with primary refractory DLBCL who demonstrate chemosensitivity to salvage chemotherapy (SC). We assessed the latter among 184 patients, 144 of whom started SC, with 84 responding and 72 receiving HDC–ASCT. The 5-year survival rate was 58.9%; the median overall survival (OS) was not reached. The difference in response to SC (partial response versus complete response) was significant, with higher 2- and 5-year OS rates in patients with CR (78.1% and 74.9%, respectively) than in those with PR (55.3% and 47%, respectively). The median OS for the whole group was 15 months and particularly patients who had progressive disease after frontline R-CHOP had dismal outcomes. Our study suggests that in patients with primary refractory DLBCL without initial progressive disease after frontline R-CHOP, the depth of response to SC before HDC–ASCT is predictive of relapse.

## Introduction

Diffuse large B-cell lymphoma (DLBCL) is the most common subtype of non-Hodgkin lymphoma. Rituximab with anthracycline-based combination frontline chemoimmunotherapy can cure 50–60% of patients.^1^ However, depending on the adverse prognostic factors from the International Prognostic Score (IPI), 20–50% of patients with DLBCL will relapse or be refractory to rituximab, cyclophosphamide, doxorubicin, vincristine, and prednisone (RCHOP) chemoimmunotherapy after achieving complete response (CR).^2^ Salvage chemotherapy and, in case of response, high-dose chemotherapy and autologous stem cell transplantation (HDC–ASCT) were the standard therapies for these groups of patients until chimeric antigen receptor T-cell therapy (CAR-T cell therapy) became available.^3^

Few studies have appraised the outcomes of patients with DLBCL who experience partial response (PR), stable disease (SD), or progressive disease (PD) (called primary refractory disease) in response to frontline R-CHOP therapy. Data for evaluation of the outcomes of HDC–ASCT therapy in patients with primary refractory DLBCL who demonstrate chemosensitivity to salvage chemotherapy are also very limited. The SCHOLAR^4^ study and Center for International Blood and Marrow Transplant Research (CIBMTR)^5^ analysis have published their results of HDC–ASCT in primary refractory DLBCL.

Here, we report the outcomes of patients with primary refractory DLBCL, herein defined as patients experiencing PR, SD, or PD in response to frontline R-CHOP therapy, and also assess the outcomes of HDC–ASCT therapy in these patients who achieve CR and PR after salvage chemotherapy.

## Materials and methods

The Institutional Research Advisory Council (RAC Number: 2021-048) approved the use of the prospective lymphoma and stem cell transplant patient database for this study. All patients and/or their guardians provided informed consent for all treatments, procedures and HDC–ASCT, as per institutional requirements.

### Patients

All patients aged 14–60 years with primary refractory DLBCL who visited the Department of Adult Medical Oncology were identified from lymphoma and stem cell transplant databases. Analog and electronic charts were reviewed to collect the required data. Before HDC–ASCT, all patients were required to have adequate hematological, renal, hepatic, pulmonary and cardiac functions. To assess the outcomes of primary refractory DLBCL as a group, patients from 2002 to 2018 were included. Patients with primary central nervous system lymphoma, with Myc-positive DLBCL (assessed by complete immunohistochemistry and fluorescent in-situ hybridization), T-cell lymphoma, relapsed mantle cell lymphoma, and follicular center cell grade 3A were excluded.

**Table 1.** Basic patient characteristics.

**Basic characteristics**			*N* = 184
**Median age, years (range)**			38 (14–60)
**Male gender, (%)**			57
**ECOG PS**			
0–1			142
≥3			42
**Upfront treatment received**			RCHOP
**Stage**			
I–II			35
III–IV			149
**IPI risk classification**			
0–1 point			25
≥2 points			159
**Basic characteristics before salvage**			
**Total number of salvages**			144
**Median age, years (range)**			38 (15-⁠61)
**Male gender, (%)**			59
**ECOG PS**			
0–1 point			112
≥3 points			32
**Stage**			
I–II			33
III – IV			111
**IPI risk classification**			
0–1 point			48
≥2 points			96
			
**Total number of HDCs and ASCTs**			72

Abbreviations: ASCT, autologous stem cell transplantation; ECOG PS, ECOG Scale of Performance Status; HDC, high-dose chemotherapy; IPI, International Prognostic Score.

### Definitions

All patients were staged according to the Ann Arbor/Cotswold’s modification staging system. Bulky disease was defined as a mediastinal mass greater than or equal to one-third of the maximum thoracic wall diameter, or any mass of 10 cm (long axis as per institutional guidelines). International Working Group response criteria were used for computed tomography (CT) and positron emission tomography (PET) scans, when available. Response to therapy was determined by the 1999 and 2007 International Working Group response criteria.^6,7^

Primary refractory disease was defined as PR, no response (NR)/SD, PD after planned multi-agent anthracycline-based chemoimmunotherapy induction with or without radiation therapy (XRT), or relapsing within 3 months of finishing the planned first-line treatment after achieving CR or unconfirmed CR (CRu).

Patients with NR/SD or PD based on CT scan ± fluorodeoxyglucose (FDG)-PET scan after the first salvage treatment with a performance status of 0–1 received the second salvage treatment. Patients achieving CR or PR to the first or second salvage treatment proceeded to HDC–ASCT. Those who progressed on the second salvage treatment or who could not proceed to HDC–ASCT were offered the best supportive care.

### Salvage chemotherapy, HDC, and stem cell mobilization

Etoposide, solu-medrol, Ara-C, and cisplatin with rituximab (R-ESHAP) were primarily used as salvage chemotherapy, and also for the mobilization regimen for stem cell collection, as previously reported.^8^ Carmustine, etoposide, cytarabine, and melphalan (BEAM) were used as HDC in all patients.

### Response and post-HDC–ASCT evaluations

Patients on primary chemotherapy had clinical and radiological response evaluation in the middle and at the end of the planned initial treatment, or as clinically indicated. All patients underwent response evaluation after 2–3 cycles of salvage chemotherapy. Patients underwent restaging imaging evaluation after HDC–ASCT at approximately 100 days (approximately 3.5 months) to check the disease status, and also when clinically required.

### Statistical methods

For transplanted patients, prognostic factor data were collected both at the time of diagnosis and before the initiation of salvage chemotherapy. For categorical variables, frequencies and percentages were used to describe the patients’ characteristics, whereas medians and interquartile ranges were used for continuous variables. The Chi-square test was used to compare categorical variables, whereas the Mann–Whitney U test was employed to examine continuous variables. Overall survival (OS) probabilities were calculated by the Kaplan–Meier method, non-relapse mortality and relapse rates were assessed using the cumulative incidence method while accounting for competing risk, and variance was calculated with the Greenwood formula. Survival curves were compared using the log-rank test. A multivariate Cox proportional hazards regression model was utilized to identify risk factors associated with OS. Using time-dependent covariates to test the proportional hazards assumption, no factors were identified as violating this assumption. Variables with a p-value of less than 5% were preserved in the final model using a forward model-building technique. It was shown that there were no interactions between the risk variables. p-values of 0.05 or less were considered significant. RStudio was utilized to perform the analysis (Version 1.4.1106, RStudio Team, PBC, Boston, MA).

### Model building

Various prognostic factors were evaluated using univariate analysis aimed at reducing the number of factors to be included in the multivariate model to avoid model overfitting. The initial multivariate model included any factor with a significance level <0.2 in the univariate analysis. The multivariate model was built using the backward method. Cumulative incidence (CI) and hazard ratios (HR) were calculated for the identified factors.

## Results

The results are reported in two sections: 1) the entire group of 184 refractory patients and 2) all refractory patients who underwent HDC–ASCT.

### Entire group of 184 refractory patients

Overall, 530 patients with DLBCL were diagnosed during 2002–2018. Of them, 184 were identified as having primary disease refractory to RCHOP therapy. The male-female ratio was 104:80, and the number of patients with stages I–II was 35 and III–IV was 149. The median age was 38.68 years (14–60 years). The median follow-up time for all patients was 15 months (1.6–242 months), and for all alive patients it was 101 months, i.e., approximately 8.5 years (22–242 months).

## Treatment outcome

Salvage chemotherapy was not considered an option in 40 (22%) patients, and they were offered supportive and palliative care only, based on clinical assessments. Salvage chemotherapy was initiated in 144 patients; 40 received more than one line of salvage chemotherapy. A total of 84 patients responded to salvage chemotherapy: 38 (26%) achieved CR, and 46 (32%) achieved PR. After salvage chemotherapy, 59 (41%) patients had PD, and one had SD. Seventy-two (50%) of 144 patients were not eligible for HDC–ASCT (59, 12, and 1 patient due to PD, co-morbidities, and patient refusal, respectively). The 72 (50%) patients who responded to salvage chemotherapy received HDC–ASCT. Of these 72, 56 (77%) responded to HDC–ASCT: 52/72 (72%) achieved CR, 4/72 (5.6%) PR, and 15/72 (20.8%) PD, with 1 patient being lost to follow-up (Table 2).

**Table 2.** Characteristics of patients with HDC and ASCT at initial presentation and survival

**Variable**	**Total patients**	**Percentage (%)**
Total patients	72	100
Median age at diagnosis (y)	31.84 (14.9–59.2)	
Male gender	48	66.7
Female gender	24	33.33
**Response to initial chemo-immunotherapy**		
CR/CRu	13	18.1
PR	22	30-6
PD	37	51.4
**Before salvage chemotherapy**		
Stages III–IV	54	75
IPI, 0 – 1 point	28	38.9
IPI, >2*	36	50
Use of ESHAP+ Rituximab	64	88.8
Disease status* before HDC – CR/CRu	33	46
Disease status before HDC – PR	38	528
PET scan available before HDC/Post salvage	55	76.4
Negative PET findings	33	45.8
**Response after HDC-ASCT**		
CR/CRu (32 with CR)	52	72.2
PR	4	5.6
PD	15	20.8
Post HDC auto-SCT radiation**	18	25
Radiation for consolidation	10	13.9
Radiation for PR eradication	3	4
Radiation for PD eradication	5	6.9
**Survival/disease status after HDC-ASCT**		
Alive/alive with no disease	42	58.3
Alive with disease	0	0
Dead	30	41.7
Died of disease	25	34.7
Died of other causes	5	6.9
**Type of treatment failure (first event)**		
Progressive disease	15	21
Persistent disease	4	5.6
Relapse post-HDC-ASCT	12	16.6
Died of other cause	3	4.2
Treatment-related mortality	2	2.8
No event	36	50

Abbreviations: CHOP, Cyclophosphamide, vincristine, procarbazine, prednisone; CR, complete remission; CRu, complete remission unconfirmed; HDC, high-dose chemotherapy; IPI, International Prognostic Score; NR, no response; PD, progressive disease; PR, partial response; RT, radiation therapy; SCT, stem cell transplantation; SD, stable disease.

*Eight patients had missing IPI values, and one patient had a missing value for the disease status before HDC because HDC was received at another institution.

**Of the 8 patients who received RT for PR/PD, 2 are alive, whereas 7 of 10 patients who received RT for consolidation are alive.

## Survival outcome

**Figure 1.**
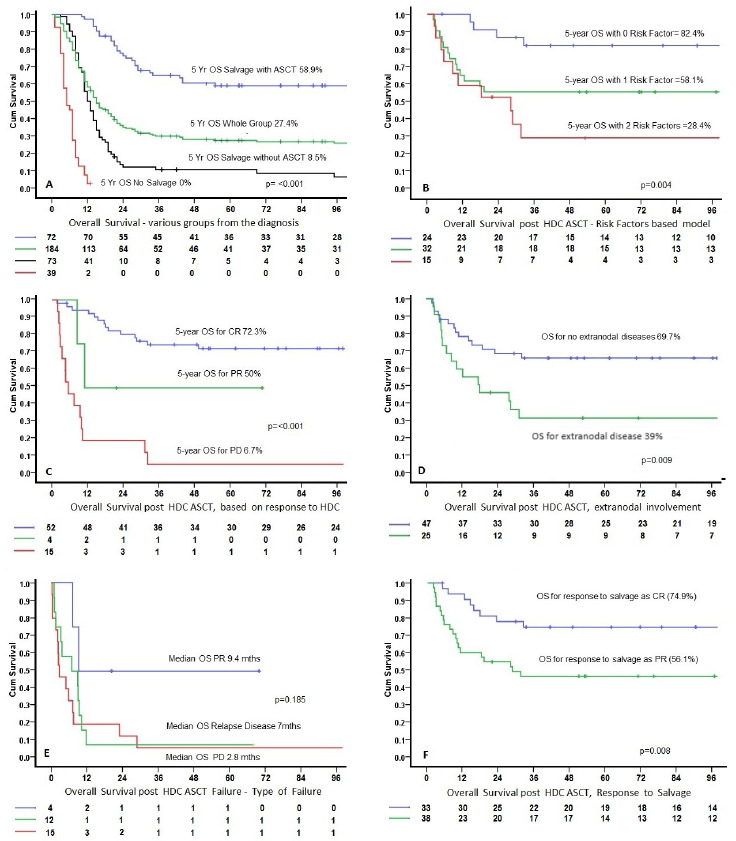
Survival curves were compared using the log-rank test. A multivariate Cox proportional hazards regression model was utilized to identify risk factors associated with OS.

The median OS for the whole group was 15 months: 48 (26.1%) were alive, 47 were alive and in remission, and 1 was alive with disease and under treatment. A total of 136 patients died (73.9%), of which 131 died of disease and 5 who had HDC–ASCT died of other causes (2 due to treatment-related mortality, 3 of other causes, and 4 of 5 were in CR at the time of death). The 5-year Kaplan–Meier-estimated survival (%) and median OS for the various groups were as follows: whole group (184 patients) 26.9% and 15 months; not treated with salvage chemotherapy (40 patients) 0% and 5 months; salvage with no ASCT (72 patients) 8.5% and 13 months; and salvage with ASCT (72 patients) 58.9% and median OS not reached. All 40 patients who did not receive salvage chemotherapy died of disease. Out of 72 patients, 66 who received salvage chemotherapy but did not receive HDC–ASCT died (Table 3, Figure 1A).

**Table 3.** Post-HDC-ASCT management and outcomes in 31 patients requiring intervention

**Variable**	**Total patients**	**Percentage (%)**
Total patients with PR, PD, or relapsed disease	31	100
Type of 1st intervention after HDC failure		
Chemotherapy alone	9	29
XRT alone	9	29
Supportive care only (No treatment)	13	42
Type of 2nd intervention after HDC failure		
Chemotherapy alone*	5	16
Chemotherapy + XRT	1	3
Supportive care only	4	13
Number of lines of treatment (101 patients)		
0 lines	13	42
1 line only	18	58
2 lines	6	19
2nd SCT (auto or allogeneic)**	3	9.6
Best response ever: post 1st HDC failure with various treatments		
CR	6	19
PD	12	38.7

Abbreviations: CR, complete response; PR, partial response; PD, progressive disease; HDC, high-dose chemotherapy; SCT, stem cell transplantation; XRT, without radiation therapy.

*Included 2 patients who received pembrolizumab and nivolumab.

**All 3 patients died; 2 with PD and 1 with CR had post-SCT complications.

At time of diagnosis, there were 17 patients (9.2%) who received RCHOP and had short CR (CR with relapse within 3 months) ; 11 (64.7%) underwent HDC–ASCT with a 2-year OS rate of 58.8% and a median OS from diagnosis of 59 months (eight are alive). PR was achieved in 41 patients (20.3%) (including 3 with SD/NR); 26 (63.4%) underwent HDC–ASCT with a 2-year OS rate of 60.6% and a median OS from diagnosis of 34 months (17 were alive). PD was observed in 126 patients (68.5%), and 35 (27.7%) underwent HDC–ASCT; the 2-year OS survival rate was 25.4%, and the median OS from diagnosis was 11 months (22 are alive) (short CR versus. PR, p=0.68; short CR versus. PD, p=0.004; PR versus. PD, p≤0.001). This suggests that the outcomes HDC-ASCT were particularly dismal for patients who had progressive disease after frontline R-CHOP

### Patients who underwent HDC–ASCT

Patients’ characteristics before the initiation of salvage chemotherapy are shown in Table 2. From 2002 to December 2018, 72 patients underwent HDC–ASCT for refractory disease. One had an allogeneic stem cell transplant due to stem cell collection failure; 12/72 patients underwent transplant after receiving third-line treatment (second salvage). The median age was 31.84 years at the time of HDC–ASCT (15.6–60 years). Nine patients were aged 14–21 years, and 29 (40%) were aged <30 years.

Pathology confirmation for refractory disease after primary chemotherapy/before salvage chemotherapy was performed in 32/72 (44.4%), whereas other patients with no biopsy had unequivocal clinical/radiological evidence of progression/refractory disease. Transplant eligibility was discussed and approved in the section’s combined stem cell transplant planning meeting.

Before February 2005, FDG-PET/CT scan was not part of our routine post-salvage/pre-HDC–ASCT evaluation. From January 2002 to December 2018, 38 out of 72 patients had FDG-PET/CT scans as a routine evaluation after initial treatment/before salvage chemotherapy. Post-salvage FDG-PET/CT scan was available in 55/72 patients. Post HDC–ASCT, 47/72 had an FDG-PET/CT scan. RESHAP was used as a mobilization regimen in 64/72 (88.8%) patients and other mobilization regimens were used in 12 patients.

#### Post-HDC–ASCT evaluation and follow-up

The median follow-up was 101 months for all alive patients (22–232 months, except for one patient who had achieved CR and was lost to follow-up at 5 months). After HDC–ASCT, 52/72 (72.2%) patients had achieved CR; 33 (46%) of these were already in CR after salvage chemotherapy (Table 2). PD was observed in 15 (20.8%) patients. A total of 42 patients (58.3%) were alive and in remission. Five patients died due to other causes: four achieved CR and one had an unknown disease status. Twelve of 72 patients underwent transplant after receiving third-line treatment (second salvage); 5/12 (42%) were alive. The impact of the response to HDC–ASCT was also evaluated for median, 2-year, and 5-year OS, with significant differences for patients who achieved CR (not reached, 80.4%, and 72.3%, respectively), PR (11 months, 50%, 50%, respectively), or PD (5.5 months, 20%, 6.7%, respectively) (all p<0.001) (Figure 1C).

#### Post HDC–ASCT failure and management

Overall, 31 patients had HDC–AST failure in the form of persistent disease/PR (4 patients), PD (15 patients), and relapsed disease after achieving CR (12 patients). Of these 31 patients, 13 (42%) were not considered for any further treatment/intervention due to poor performance status or rapid PD. The remaining 18/31 patients (58%) were offered various treatments as shown in Table 3 and their survival was as in Figure 1E.

### Prognostic factor analysis and model building

Seventeen prognostic factors, before the initiation of salvage chemotherapy, were evaluated with univariate analysis as shown in Table 4. Multivariate analysis was performed for the final model for OS, identifying response to salvage treatment (PR versus CR) and the presence versus absence of extranodal diseases as significant factors. Regarding the response to salvage treatment, for PR (56.1%) versus CR (74.9%), the HR was 2.5 (95% confidence interval 1.09–5.72) (p=0.008). In terms of the presence (39%) versus absence of extranodal diseases (69.7%), the HR was 2.35 (95% confidence interval 1.11–4.95) (p=0.005). The model with these two risk factors showed that the 5-year OS with 0 (82.4%), 1 (58.1%), and 2 (28.4%) factors were significantly different (p≤0.004) (Figures 1B, 1D, and 1F).

**Table 4.** Kaplan–Meir overall survival estimates of various prognostic factors before the start of salvage therapy using univariate, multivariate analysis, and a score-based survival model

**Univariate analysis**					
Covariates*	**Total patients**		**2-year OS %**	**5-year OS %**	**p-value**
Female gender Male gender	24 48		74.1 60.3	65 56	0.55
<30 years >30 years	29 43		62.1 66.8	55 61.7	0.59
Performance status, >2 Performance status, 0–1	19 53		52.6 69.3	46.8 63.3	0.15
Stages before salvage, III–IV Stages before salvage, I–II	54 17		66.2 58.8	58.3 58.8	0.83
Bone marrow, involved Bone marrow, uninvolved	9 59		44.4 65.6	33 60	0.14
Extranodal disease, present Extranodal disease, absent	47 25		52 71.9	39 69.7	0.009
Spleen involvement, yes Spleen involvement, no	9 59		44.4 65.6	33 60.2	0.1
Bulky disease, yes Bulky disease, no	14 56		64.3 65.6	64.3 58	0.64
Mediastinal involvement, yes Mediastinal involvement, no	28 39		60.7 65.9	50 63.2	0.47
Age-adjusted IPI >2 Age-adjusted IPI 0 – 1	36 33		51.8 75.8	45.7 69.4	0.019
B symptoms, present B symptoms, absent	18 53		50 69.3	44.4 63.3	0.154
Response to salvage, no CR Response to salvage, CR	38 33		55.3 78.1	47 74.9	0.008
PET response to salvage, no CR PET response to salvage, CR	23 29		56.5 75.9	56.5 72.2	0.099
CT response to salvage, no CR CT response to salvage, CR	55 9		63.6 100	56.1 100	0.028
Type of refractoriness PD/SD PR Short CR	35 26 11		25.4 60.6 58.8		0.97
**Multivariate analysis–overall survival**					
	Total patients	95% CI** range		HR (OS)	p-value
Extranodal disease – present Extranodal disease – absent	25 46	1.11–4.95		2.35 1	0.005
Response to salvage – no CR Response to salvage – CR	38 33	1.09–5.72		2.5 1	0.008
Prognostic model and overall survival prediction based on risk factors***					
Number of factors	Total patients	2-year %		5-year %	Median – months
0	24	87		82.4	Not reached
1	32	56.3		58.1	Not reached
2	15	53.3		28.4	28.2

Abbreviations: CI, confidence interval; CR, complete response; HR, hazard ratio; LDH, lactate dehydrogenase; PET, positron emission tomography; PD, progressive disease; OS, overall survival; SD, stable disease.

*Some variables had missing values, and the total is not 72.

**HR, confidence interval, Lower–upper limits (CI).

***Based on 3 factors identified in multivariate analysis.

### Impact of remission status before HDC–ASCT

As shown in Table 4, following salvage chemotherapy, a higher proportion of patients had achieved PR (38 patients [52.8%]) before HDC–ASCT, compared with CR (33 patients [46%]). Survival analysis confirmed that patients who achieved CR before salvage chemotherapy can experience durable disease control with HDC–ASCT, with significantly higher 2-year and 5-year OS rates for CR patients (78.1% and 74.9%) than for PR patients (55.3% and 47%) (p=0.008).

## Discussion

Our study suggests that the depth of response to salvage chemotherapy is predictive of relapse after HDC–ASCT in primary refractory DLBCL. Moreover, our findings showed that patients with primary refractory DLBCL after frontline chemoimmunotherapy can experience durable disease control with HDC–ASCT if they are eligible, respond to salvage therapy, achieve CR and, possibly, may not require CAR-T cell therapy. However, the efficacy of HDC–ASCT in primary refractory DLBCL patients who achieve PR after salvage chemotherapy remains controversial.

Response to upfront chemoimmunotherapy is a well-known prognostic indicator for progression-free survival (PFS) and OS in patients with DLBCL. Vardhana et al.^9^ reported the outcomes of primary refractory DLBCL from a single institution, wherein only 50% of the patients responded to salvage chemotherapy. The SCHOLAR-1^4^ study is one of the largest multicenter retrospective studies that included 636 patients from different international databases, and pooled analysis was performed to assess the outcome in patients with DLBCL in various refractory settings. The objective response rate to salvage chemotherapy was 26% (CR, 7%). If we subdivide the SCHOLAR study patients into various subgroups, the median OS was higher among those who had undergone HDC–ASCT (14.4 months) than among those who did not (5.1 months). Patients who achieved CR after the last salvage chemotherapy had longer survival (median OS, 14.9 months) than the non-responders (median OS, 4.6 months). Those who achieved CR underwent HDC–ASCT, and their median OS was more than 6 years. Of the patients who achieved PR and underwent ASCT, the median OS was 17.8 months, suggesting that a response to salvage therapy is an important prognostic factor for long-term survival among relapsed/refractory DLBCL. For the patients who were unable to achieve CR or PR and who underwent ASCT after receiving an intervening line of therapy, the median OS was 8.7 months.

A review of the CIBMTR database^5^ also highlighted the response to salvage therapy as an important prognostic factor for long-term survival in primary refractory DLBCL. In that study, in patients with primary refractory DLBCL after frontline RCHOP, HDC–ASCT resulted in durable disease control in those with chemosensitivity to salvage therapy. They reported that the 4-year PFS was 39% for the CR versus 43% for the PR group (p=0.69). At 4 years, the OS was comparable at 50% in the CR versus 49% in the PR group (p=0.8).

After the CIBMTR database review,^5^ Shadman et al.^10^ compared the outcomes of patients with DLBCL treated with HDC–ASCT versus CAR-T therapy in who achieved PR after salvage chemotherapy. They observed that the cumulative incidence of relapse/progression was lower with HDC–ASCT at 2 years (40% versus 52%, p=0.05). The 2-year OS rate was higher in the HDC–ASCT group than in the CAR-T group (69% versus 47%; p=0.004). This clearly shows that HDC–ASCT is associated with a similar PFS rate, but a lower incidence of relapse and an improved OS, compared with CAR-T cell therapy.

Our center is a major tertiary care center in the Kingdom of Saudi Arabia performing around 250 stem cell transplants annually. Patients with relapsed or refractory disease at other institutions are referred here for HDC–ASCT. Due to this referral bias, almost 50% of our patients with lymphoma have undergone HDC–ASCT for refractory disease.^8^

In our study, if we assess the depth of response to salvage chemotherapy and the outcome, the 2- and 5-year OS for CR patients (78.1% and 74.9%, respectively) were significantly higher than those for PR patients (55.3% and 47%, respectively; p=0.008). These results are comparable to the data presented by the CIBMTR^5^ group and Shadman et al.,^10^ signifying that in patients with primary refractory DLBCL who achieve CR after the salvage regimen, HDC–ASCT should still be the standard of care. In contrast, the data are less consistent for patients who only achieve PR after salvage chemotherapy, and they are potential candidates for CAR-T cell therapy. Patients who did not respond to salvage therapy or were not able to receive ASCT had particularly poor outcomes.

The introduction of CAR-T cell therapy as a therapeutic option for patients with relapsed DLBCL has been a major development in lymphoma treatment. Both the ZUMA-7^11^ and the TRANSFORM^12^ trials showed the superiority of a CAR-T cell product over the standard of care (HDC–ASCT) in the relapsed or refractory setting. However, the relative effectiveness of HDC–ASCT versus CAR-T therapy in patients with primary refractory DLBCL who achieve CR or PR after salvage chemotherapy is not known. CAR-T cell therapy is expensive, involves a complex engineering process and is accessible only at specialized centers. In real-world practice, it is unlikely for patients to have immediate access to such specialized therapy, and many physicians start salvage chemotherapy while they prepare for CAR-T cell therapy. Recent data from the CIBMTR^5^ and from Shadman et al.^10^ indicate that outcomes with HDC–ASCT are perhaps better than those with CAR-T cell therapy in patients who show a response to salvage chemotherapy, especially in those who have CR to salvage chemotherapy.

The strengths of our study include the fact that it is the largest single-center study from the Middle East before CAR-T cell therapy was approved at our local center. Additionally, all patients received uniform first-line and salvage chemotherapies with a uniform practice for selecting transplant-eligible patients.

Our study has some limitations, including its retrospective nature and the absence of some important variables, for example, cell-of-origin status. Furthermore, our findings are based on a small group of patients with primary refractory disease who afterward demonstrated chemosensitivity to salvage therapy and were candidates for consolidation with HDC–ASCT. We understand the importance of PET/CT for response assessment. Before February 2005, FDG-PET/CT scan was not part of our routine post-salvage/pre-HDC–ASCT evaluation.

In conclusion, our study suggests that in patients without initial progressive disease after frontline R-CHOP, the depth of response before HDC–ASCT remains predictive of relapse in a primary refractory DLBCL.

### Author contributions

Conceptualization: M Shahzad Rauf (Lead), Saad Akhtar (Equal). Data curation: M Shahzad Rauf (Lead), Irfan Maghfoor (Equal), Muhammad Aseafan (Equal), Khadijah Al Shankati (Equal), Ali M. Alhanash (Equal), Faateh Sohail (Equal), Saad Akhtar (Supporting). Formal Analysis: M Shahzad Rauf (Lead), Tusneem A. M. Elhassan (Lead), Saad Akhtar (Supporting). Investigation: M Shahzad Rauf (Lead). Writing – original draft: M Shahzad Rauf (Lead). Writing – review & editing: Irfan Maghfoor (Equal), Muhammad Aseafan (Equal), Khadijah Al Shankati (Equal), Ali M. Alhanash (Equal), Faateh Sohail (Equal), Saad Akhtar (Equal).

### Disclosure of interest

The authors declare that they have no conflict of interest.

### Ethics approval

This study was approved by the Institutional Research Advisory Counsel.

### Data availability statement

All data supporting the findings of this study are available within the paper and its Supplementary Information.
